# Understanding the meaning of autonomy in adolescent pregnancy decision-making: lessons from Ghana

**DOI:** 10.11604/pamj.2021.40.34.29220

**Published:** 2021-09-13

**Authors:** Luchuo Engelbert Bain

**Affiliations:** 1 Lincoln International Institute for Rural Health (LIIRH), University of Lincoln, Lincoln, United Kingdom,; 2 Global South Health Services and Research, GSHS, Amsterdam, The Netherlands

**Keywords:** Ethics, reproductive health, adolescents, Ghana, pregnancy

## Abstract

**Introduction:**

adolescent pregnancy in Ghana, like in most low and middle income countries, is an issue of immense public health importance. Pregnant adolescents are faced with the stronger dilemma of either terminating the unwanted pregnancy or keeping it. This discourse which is based on findings from empirical research in Accra Ghana aims at contributing to the usefulness of understanding the meaning and scope of autonomy when it comes to providing ethically grounded, and adolescent friendly, reproductive health care services to pregnant adolescents. The aim of this work was to document the meaning and determinants of autonomous decision making among pregnant adolescents in the James Town area of Accra, Ghana.

**Methods:**

thirty (30) semi-structured in depth interviews were conducted among adolescents who had been pregnant at least once, 23 in depth interviews among purposively selected stakeholders (parents, teachers, NGO staff working in reproductive health, community volunteers), and 8 focus group discussions among parents, teachers, adolescent students who had not been pregnant before, and adolescents who had at least one pregnancy in the past. Data were transcribed verbatim and analyzed thematically.

**Results:**

most adolescents reported that the final decision to continue a pregnancy to term or go in for an abortion was taken by them. The partner´s willingness to take responsibility of the pregnant adolescent and baby, as well as financial considerations, were main players in deciding upon the pregnancy outcomes. Cultural desirability for children and health care provider/father paternalism (power dynamics) in the decision-making process were central considerations in the decision-making process. Unaffordable and unfriendly safe abortion services pushed adolescents to either continue pregnancies to term against their will, or opt to visit unsafe abortion care providers.

**Conclusion:**

adolescents stand to make truly autonomous decisions if they are provided with the right information, at the right time, at the right place, by the right persons, and in the right way. Health system, economic, and cultural factors play significant roles in rendering pregnant adolescent autonomy meaningful when deciding upon their pregnancy outcomes. Continuing pregnancies to term against one´s will or being forced to go in for an abortion are ethically unjustified. Further research is required to examine the long-term consequences of forced pregnancy terminations or births.

## Introduction

The global prevalence of adolescent pregnancy (first pregnancy before the age of 18) stands at 19%. This prevalence is highest in the sub-Saharan African region (19.3%) and lowest in North Africa (9.2%) [1]. It is estimated that by 2030, approximately 1 in every 4 adolescent girls will live in sub-Saharan Africa [2]. The prevalence of ever being pregnant ranges between 16% and 19% among 15-19-year-old adolescents in a multi-country study in disadvantaged urban settings in Baltimore (USA), Johannesburg (South Africa), Ibadan (Nigeria), Delhi (India) and Shanghai (China) [3]. Prolonging sexual debut and staying in school were factors found to correlate with a lower number of adolescent pregnancies in this study. In Ghana, adolescents make up 22% of the total population. About 30% of births registered in Ghana in 2014 occurred among adolescents (18 years or younger) [4]. Providing adolescent-friendly counseling services improves their utilization of sexual and reproductive health services [5]. However, training providers in youth-friendly service provision is not sufficient on its own to increase service use. Targeted school-based programmes and community outreach activities have been shown to be effective complements in improving the use of reproductive health services by adolescents [6].

In general, the participation of the patient/client in decision-making is increasingly being regarded as legally, ethically (autonomy and self-determination) and socially desirable [6, 7]. In a qualitative study that explored the decision-making experiences of 28 female abortion seekers aged between 15 and 30 years in Cape Coast in Ghana, the participants claimed autonomy in their abortion decisions [8]. Previous findings from our own study of in-depth interviews with adolescents who had either continued a pregnancy to term or sought an abortion in Jamestown in Accra also reported that adolescents claimed the responsibility of having made the final decision themselves [9]. The inadequacy of the comprehensive sexuality education packages (“sex” avoiding), prevalent myths regarding modern contraception, social and health provider-associated stigma, and lack of trust in the health care providers are barriers to obtaining optimal levels of information regarding adolescent sexuality, pregnancy, and pregnancy resolution options that have been reported in Ghana [8-10]. An ideal autonomous decision (informed) will require the agent to have complete, relevant and accurate information for all available options [6, 7].

Sociocultural and emotional factors influence the decisions made [6]. A retrospective mixed-method study among 401 women who had abortions in Ghana found that the friends, mothers, and male partners influenced the final decision to various extents [10]. Challa *et al*. reported the positive and negative roles that partners, peers and parents play in adolescent sexual and reproductive health decision-making in Ghana, especially regarding sexual debut, contraceptive use and pregnancy resolution [11]. In this qualitative study of 63 interviews with young women aged between 18-24 years in Accra and Kumasi revealed the following: respondents reported having been forced to initiate sex by peers and partners: parents forced girls into prostitution to support the family: the community showed negative attitudes towards the use of contraception by adolescents: the health facilities were not supportive enough to encourage service use: and the religious considerations were against use of contraception, especially among single adolescents. Protective parental and community factors have also been reported, such as encouraging the postponement of sexual intercourse and concentrating on studies when young [11].

The Ghanaian abortion laws, as well as the Ghana Health Service (GHS) guidelines on reproductive health, fail to provide any directives specific to counselling and decision-making for pregnant adolescents. Regarding this gap, it is important to investigate the meaning of autonomous decision-making among pregnant adolescents. A better understanding of autonomous decision-making could contribute to the establishment of respectful and adolescent-friendly health services. The aim of this paper was to document the extent and determinants of autonomous decision-making among pregnant adolescents in the James Town area of Accra, Ghana.

## Methods

### Study design and data collection approaches

This study adopted a qualitative study design, grounded mainly on in depth interviews and focus group discussions. Semi-structured, in-depth interviews were carried out among 15 teenage mothers and 15 adolescents who had had at least one abortion, in the Accra area (Ghana). The working hypothesis was that 30 interviews were sufficient to allow for data saturation. A vignette-based focus group discussion design was adopted to investigate the risk factors of early adolescent pregnancies (< 15 years old). Adolescents were recruited with the aid of a local NGO, Act for Change Ghana that has been working in sexual and reproductive health programs in the area for over 15 years and the Adolescent Health Unit of the USSHER Polyclinic in James Town. Permission was obtained from the Principals of the key secondary schools in the area (Sacred Heart Technical Institute Secondary school and the Bishop Girls´ primary school) to approach teachers to participate in the study and students that had never been pregnant before. To obtain a broad range of perspectives from actors not directly involved in the decision-making process, 8 focus group discussions were carried out among several purposively selected groups of participants: parents, teachers, adolescent students who had not been pregnant before, and adolescents who had had at least one pregnancy in the past. The vignette was a hypothetical case of a 15-year-old high school student, who had not seen her menses for the past 6 weeks. Interviews among 23 purposively selected stakeholders including parents, teachers, and NGO staff working on reproductive health, and community volunteers were carried out. The study was carried out between January and March 2018.

### Bias

To address issues that could result from selection bias, a variety of stakeholders was selected to obtain diverse opinions. Vignette based focus group discussions were used to avoid receiving socially acceptable responses.

### Theoretical frameworks for an understanding of autonomy

Principlism, as introduced by Beachamp and Childress, has been the dominant decision-making model, especially in clinical medicine [7]. The authors outlined four main principles: autonomy, justice, beneficence and non-maleficence. Their influential definition identifies autonomous decisions as those made intentionally, with substantial understanding and free from controlling influences. They explicitly excluded people who are not 'competent' to make independent decisions from the protection of the principle of respect for autonomy [7]. The 6 phase thematic analysis approach proposed by Braun and Clarke in analyzing the data was used [12]. This was to allow for a holistic capturing of the different dimensions of autonomy from empirical data and properly connecting our findings to the theoretical frameworks chosen. Principlism has lately been criticized as inadequate in decision-making in health care, especially in situations in which the clinical encounter needs to take into consideration the sociocultural realities of the agents. In their critique on principlism, Clouser and Gert link the contradictory nature of principles and inadequate treatment of moral relativism. The authors argue that these “principles” do not function as claimed, and that their use is misleading both practically and theoretically since the “principles of Beauchamp and Childress” lack any systematic relationship to each other, and they often conflict with each other [13].

### Kant, strong duties

Immanuel Kant (1724-1804) argued that the supreme principle of morality is a standard of rationality that he dubbed the “Categorical Imperative” (CI) [14]: an unconditional requirement that must be obeyed in all circumstances and is justified as an end in itself. It is best known in its first formulation: Kant held that the fundamental test for a strong moral principle that has the potential to guide our moral duties is the three-fold question posed by the categorical imperative. It is categorical in virtue of applying to me and everybody else unconditionally. It does not apply to us, in other words, when we have antecedently adopted some personal goal for ourselves. Three issues were important to Kant in ethical decision-making: autonomy in the sense of being able to make an autonomous decision (well informed, free from coercion and non-rational inclinations), the categorical imperative in the sense of duty and intention, and respect for others. A rational analysis from the adolescent, parents, partner or health care provider perspectives could lead to different rational “moral” conclusions. Contextual analyses attempt to explain both the positive and negative implications of social relationships on individuals´ autonomy [15]. These understandings support recognition of the value of good patient-health professional relationships and can optimize the particularities of the principle of autonomy in the context of the adolescent and the health provider. In pregnant adolescents, for instance, the religious values of the health care professional can bias the quality of the information provided to the adolescent regarding pregnancy termination. The decision taken, even if done by the adolescent, will not be considered autonomous as it fails to be a well-informed decision [7, 8, 10].

### Data analysis

The interviews and Focus group discussions were recorded and transcribed into English and validated by an experienced qualitative researcher. The data were anonymized before analysis. A common coding frame was developed in ATLAS.ti© 8 for Windows using open coding. The data were then analyzed using a thematic analysis approach [12]. The 6 phase thematic analysis approach proposed by Braun and Clarke in analyzing the data was used [12]. These phases are: familiarization with the data, generating initial codes, searching for themes, reviewing themes, defining and naming themes, and producing a report. The codes were applied to different blocks of the texts, and the transcripts were reviewed iteratively. The initial coding process was open, and followed by an axial coding to establish meaningful connections among emerging themes.

### Ethical considerations

Ethical approval was obtained from the Ethics Review Committee of the Ghana Health Service (GHS-ERC: 003/07/17). The study protocol was also assessed and registered by the Scientific Quality Committee of the Vrije Universiteit Amsterdam-Netherlands (EMGO+; WC2017- 025). Prior to registering their consent, all respondents were informed of study aims, measures taken for data privacy and confidentiality, as well as their rights as participants. Participants either signed a consent form or, in the case of minors, assent was obtained. Informed consent was obtained from all participants and written informed consent was obtained from a parent or guardian for participants under 16 years old.

### Ethics approval and consent to participate

This research was carried out in adherence with the declaration of Helsinki guidelines regarding research on human research subjects. Ethical approval was obtained from the Ethics Review Committee of the Ghana Health Service (GHS-ERC: 003/07/17). The study protocol was also assessed and registered by the Scientific Quality Committee of the Vrije Universiteit Amsterdam-Netherlands (EMGO+; WC2017-025). Prior to registering their consent, all respondents were informed of study aims, measures taken for data privacy and confidentiality, as well as their rights as participants. Written informed consent was obtained from a parent or guardian for participants under 16 years old. All obtained data were anonymized in order to protect the identity of the study participants. The data were stored in a safe folder, and was only accessible to the principal investigator and supervisors. Audio files from the interviews were deleted after the transcripts were made. All research data consequently presented as scientific articles or reports have no identifiable features of the study participants.

### Consent for publication

All participants have provided their consent for the publication of this manuscript.

### Availability of data and material

The data sets used and analyzed during this current study are freely available from the corresponding author.

## Results

All persons approached for the study accepted to participate giving a response rate of 100%. Almost of the respondents (95%) of were Christians.

### Decision-making experience

Adolescents who had experienced an abortion in the past reported the involvement of their parents, partners, and trusted aunts (significant others) in taking the final decision. *“During the pregnancy, my mother and siblings asked me to go in for an abortion. I had to leave home to stay with my friend´s mother till I gave birth*” [Adolescent mother #4; 17 years old].

*“My dad told me he would disown me if I didn´t get an abortion”*[Adolescent with an abortion experience #8, 17 years old].

Only two of the 15 adolescents who had an abortion used a qualified health care professional. The relationship with the partner was the most important consideration in taking the final decision.

*“I was compelled to go in for an abortion because the guy who impregnated me ran away”*[Adolescent with abortion history #10, 18 years old].

When asked who took the final decision in either continuing the pregnancy to term or opting for a pregnancy termination, 28 out of the 30 interviewed adolescents reported that the final decision was taken by themselves [9]. Though they all lived in a very religious community, religiosity was not a key consideration in making the final decision. Surprisingly, adolescent mothers who had been pregnant before and participated in the reflection on the hypothetical pregnancy outcome decision-making (vignette) still proposed the girl´s father as the final decision-maker.

### Who decides, who should decide?

Despite sometimes opposing views from external influencers, most (28/30) adolescents felt the final decision was an individual one, emphasizing their autonomy. Supporting quotes regarding preferences on who should take the final decision are summarized in Table 1. Peer opinion did not seem to discourage adolescents who opted for abortions. Health care personnel leaned towards the respect of adolescent autonomy in final decision-making.

**Table 1:** who should take the final decision and why?

Who should take the final decision?	Supporting quotes
The father	The father has the final decision; He´s the only person who can say the final thing to either make me deliver or terminate it because he´s the family head [JHS1]
	The dad is the only person who has that authority to say I should give birth or terminate it because we have his name on us and besides, he´s the head of the family so all of us before we do something we ask his permission before we make the move [JHS 8]
	The girl´s father has the final say as to whether his daughter aborts the pregnancy or not. It is not the question of whether he caters for his daughter or not. The boy who impregnated the girl should not come in at all because he has no right first of all to sleep with the girl to the point of making her pregnant [Parent 3].
The mother	Mothers have got a big role to play in the decision-making process. Mothers actually decide on the fate [Parent , M, 5]
	It is her mother who has the final say with her daughter´s pregnancy [JHS6]
The two families	I think the parents from both sides should come together to agree on how best they can solve the problem [Parent, F, 10].
The girl herself	She knew that becoming pregnant could arise from having sex, so she should know what to do with the pregnancy if she gets pregnant. [Teacher, M, 2]
The partner	Yes, I think that she should let her boyfriend know about it because he is the one that impregnated her, if she doesn´t tell him, and the pregnancy is advanced before she tells him, he will deny the pregnancy. [Teacher, M, 5]
	The one who has the final say is the man who impregnated the girl, whatever he says should be final. [Parent, F, 4]
	She needs her partner to sit by her to make the decision to abort it or keep it. [Teacher, F, 6]

*“Oh definitely! I mean when it comes to age and especially when people are below the ages of eighteen, you are considered a minor. You would definitely need the help of another person. But then again, the final decision should come from you*” [Nurse #4].

Other stakeholders on the other hand, including lawyers, parents, and teachers, felt adolescents were immature, inexperienced and not capable of making rational decisions. One adolescent mother was motivated to carry the pregnancy to term in order to continue her family lineage “I am the only child to my mother, so I had to give birth to continue our lineage” [Adolescent mother #11, 18 years old].

## Discussion

It is important to highlight that the medical ethical principles involved in deciding upon the pregnancy outcome inherently medicalize adolescent pregnancies. It is true that most pregnancies among adolescents are unintended [1], but it remains unclear how and why personal pregnancy decision-making is similar to or different from medical decision-making. Medical decision-making by health professionals includes the ability to understand the medical issue, weigh the risks and benefits, appreciate the consequences of choices, and make a voluntary choice based upon understanding of the information [7, 16, 17]. The American College of Obstetricians and Gynecologists has proposed 7 steps in ethical decision-making for health care professionals [18]: 1) Identify the decision-makers; 2) Collect data, establish facts; 3) Identify all medically appropriate options; 4) Evaluate options according to the values and principles involved; 5) Identify ethical conflicts and set priorities; 6) Select the option that can be best justified; 7) Reevaluate the decision after it is acted on.

Most adolescents have sufficient cognitive capacity and emotional maturity to make many health care decisions. Research suggests that around the age of 14 to 15 years, adolescents make health-related decisions similar to those that adults make in controlled decision-making situations [19, 20]. Pregnancy in early adolescence carries with it specific considerations which are in some ways different from pregnancy in an adult. For instance, physical and emotional immaturity, lack of financial autonomy, inexperience in dealing with emotionally charged challenges (like an unintended pregnancy) [21]. Decision-making in pregnancy could be different from decision-making in other medical conditions as not only the woman is affected; the unborn fetus is involved, as are the partner, family, sociocultural attitudes towards pregnancy and abortions, legal framework, health system setup, and health provider attitudes towards abortions [9, 11].

A complete disregard to the adolescent´s capacity to appreciate the stakes associated with her pregnancy or her passive role in the decision-making process is unjustified and untrue in adolescent pregnancy resolution in Jamestown. Almost all adolescents (28/30) reported having taken the final decision to either continue the pregnancy to term or go in for a pregnancy termination by themselves, which is of the utmost relevance for policymakers [9]. Added to this, not all reported pregnancies among these adolescents were unintended. Reasons to justify intended adolescent pregnancies in this community were: the desire to feel like a woman (social acceptability of early motherhood) and a means of guaranteeing a constant source of income from the impregnating partner. For a decision to be considered autonomous, the agent needs to be well-informed regarding the potential consequences for each of the available options. It therefore follows that though most adolescents had a strong desire to take the final decision regarding their health, they lacked the correct information needed to inform their decisions. The overwhelmingly reported desire of the girl´s father to be the final decision-maker in the hypothetical case presents a clear difference between the experienced decision-making and the perceived decision-making in adolescent pregnancy resolution. Indeed, even adolescent mothers still suggested the girl´s father is the one taking the final decision for the 15-year-old hypothetical pregnant girl. Table 2 summarizes the multilevel factors involved in adolescent pregnancy decision-making.

**Table 2:** summary of multi-level factors influencing decision making in adolescent pregnancy

**Individual factors (Intrapersonal)**	Knowledge and awareness of sexual and reproductive health issues
	Cost of safe abortion fees
	Socioeconomic status
	Awareness of the abortion law (performance in school, desire to pursue studies)
**Interpersonal factors**	Quality of relations with and support from family, friends, schoolteachers
	Partner responsibility
	Peer pressure
**Organizational factors**	Cost of safe abortion fees
	Abortion stigma
	Trust in health care providers
	Friendliness of adolescent health services (privacy, confidentiality, respectful care)
	Quality of pregnant adolescents´ counselling packages
**Community factors**	Religious considerations
	Area of residence (James Town neighbourhood)
	Attitudes towards abortions, adolescent pregnancy, and adolescent childbearing
	Exposure to and use of social media platforms
	The father´s place in the family and power
**Policy/environmental factors**	Awareness regarding the abortion law
	Cost of safe abortion fees
	Comprehensive sexuality education policy

Health care providers need to understand the wider context of adolescent motherhood during the counseling process. Indeed in our setting, the health care providers should recognize that some of the adolescent pregnancies are actually intentional. It must be recognized that adolescence is a transitional stage in which physical, psychological and social changes take place that are biologically based and culturally shaped. This is an important issue, when the consent and involvement of parents (and guardians) are considered, since the degree of autonomous decision-making is considerably varied across cultures and stages of adolescence. Failure to recognize this reality might unconsciously lead to directive counseling on the part of the health care providers (assuming all pregnancies are unintended). Indeed, a consequence could be a feeling of obligation or pressure perceived by the pregnant adolescent, which can undermine a healthy adolescent-health care provider relationship and trust.

A reductionist view of autonomy limited to one agent (the pregnant adolescent) could jeopardize her life and relationships, especially when cultural forces are created and perpetuated during and after the decision-making process. Allowing the adolescent to make a final decision will respect her procreative autonomy and right to self-determination. However, taking into account the presence of the network around the adolescents, the inclusion of their preferred significant others in the decision process must be recognized and carefully acted upon. Health care providers have to be sensitized to the imperatives of providing non-directive counselling services, as well as applying high confidentiality standards in the process. Improving adolescent health-seeking practices requires the creation of an atmosphere of trust between the health care provider and the client. When health care providers offer confidential and respectful care for adolescents, they should also give them the opportunity to learn how to interact with clinicians and become responsible for their own health care. Considering the strong social ties between the pregnant adolescent and the family or friends, she should be guided to understand herself and distinguish peer coercion and hormonal drives from what is wise for her and all other adolescents in the world.

### Limitations

Conclusions derived from this work are based on empirical findings from the James Town community in Accra. Generalization of these findings to the whole of Ghana is not welcome as many sociocultural factors strongly influenced adolescents´ decision-making preferences.

## Conclusion

Despite the overwhelming expression by the adolescents with a pregnancy experience of a feeling of having taken the final decision to either terminate a pregnancy or continue it to term, these decisions were not truly autonomous. Autonomous decision-making could be reinforced in this context through context (culturally) sensitive comprehensive sexual education packages, to enhance informed decisions. The cultural context which places the girl´s father at the center of authority, even when the sexuality issues of his children are concerned, needs to be further explored. Policy makers should be aware of the fact that not all adolescent pregnancies are unintended. This could be central in the respectful counseling process of the pregnant adolescent. As ethical principles might conflict at some point in the decision-making process, health care providers should be trained in ethical decision-making, e.g. using the seven-step model [18] proposed by the American College of Obstetricians and Gynecology. This could be central in advancing respectful care for pregnant adolescents.

### What is known about this topic

- Adolescent pregnancy remains a public health concern on low and middle income countries, who decides and who should is a subject of controversy;
- Autonomy remains a key subject of debate in the ethics of adolescent pregnancy decision making;
- Autonomous decision in sexual and reproductive health constitutes a tenet of respectful care.

### What this study adds

- Cultural desirability for children and health care providers/father paternalism (power dynamics) in the decision making process were important considerations in the decision making;
- Unaffordable and unfriendly safe abortion services pushed adolescents to either continue pregnancies to term against their will, or opt to visit unsafe abortion care providers;
- Continuing pregnancies to term against one´s will or being forced to go in for an abortion are ethically unjustified.

## References

[1] Kassa GM, Arowojolu AO, Odukogbe AA, Yalew AW (2018). Prevalence and determinants of adolescent pregnancy in Africa: a systematic review and meta-analysis. Reprod Health.

[2] UNFPA Adolescent Pregnancy [Internet].

[3] Brahmbhatt H, Kågesten A, Emerson M, Decker M, Olumide A, Ojengbede O (2014). Prevalence and Determinants of Adolescent Pregnancy in Urban, Disadvantaged Settings across Five Cities. J Adolesc Health.

[4] Yussif A-S, Lassey A, Ganyaglo GY, Kantelhardt EJ, Kielstein H (2017). The long-term effects of adolescent pregnancies in a community in Northern Ghana on subsequent pregnancies and births of the young mothers. Reproductive Health.

[5] Denno DM, Hoopes AJ, Chandra-Mouli V (2015). Effective strategies to provide adolescent sexual and reproductive health services and to increase demand and community support. J Adolesc Health.

[6] In Uganda, Sexual and Reproductive Health Services and Information Fall Short for Adolescents [Internet]. Guttmacher Institute.

[7] Beauchamp TL, Childress JF (2008). Principles of Biomedical Ethics.

[8] Oduro GY, Otsin MNA (2014). “Abortion-It Is My Own Body: Women´s Narratives About Influences on Their Abortion Decisions in Ghana”. Health Care for Women International.

[9] Bain LE, Zweekhorst MBM, Amoakoh-Coleman M, Muftugil-Yalcin S, Omolade AI-O, Becquet R (2019). To keep or not to keep? Decision making in adolescent pregnancies in Jamestown, Ghana. PLOS ONE.

[10] Kumi-Kyereme A, Gbagbo FY, Amo-Adjei J (2014). Role-players in abortion decision-making in the Accra Metropolis, Ghana. Reprod Health.

[11] Challa S, Manu A, Morhe E, Dalton VK, Loll D, Dozier J (2018). Multiple levels of social influence on adolescent sexual and reproductive health decision-making and behaviors in Ghana. Women Health.

[12] Braun V, Clarke V (2006). Using thematic analysis in psychology. Qualitative Research in Psychology.

[13] Clouser KD, Gert B (1990). A critique of principlism. J Med Philos.

[14] Sullivan RJ (1979). An Introduction to Kant´s Ethics, New York: Cambridge University Press, 1994 Flew A, A Dictionary of Philosophy (2nd ed), New York St Martin´s.

[15] Entwistle VA, Carter SM, Cribb A, McCaffery K (2010). Supporting patient autonomy: the importance of clinician-patient relationships. J Gen Intern Med.

[16] Puri K, Malek J, de la Uz CM, Lantos J, Cabrera AG, Frugé E (2019). Allowing adolescents to weigh benefits and burdens of high-stakes therapies. Pediatrics.

[17] Oppong-Darko P, Amponsa-Achiano K, Darj E (2017). “I Am Ready and Willing to Provide the Service. Though My Religion Frowns on Abortion”-Ghanaian Midwives´ Mixed Attitudes to Abortion Services: A Qualitative Study. Int J Environ Res Public Health.

[18] ACOG Ethical Decision Making in Obstetrics and Gynecology [Internet].

[19] Reyna VF, Farley F (2006). Risk and Rationality in Adolescent Decision Making: Implications for Theory, Practice, and Public Policy. Psychol Sci Public Interest.

[20] Kwak Y, Payne JW, Cohen AL, Huettel SA (2015). The rational adolescent: Strategic information processing during decision making revealed by eye tracking. Cognitive Development.

[21] Institute of Medicine National Research Council (2019). Read “Adolescent decision making: implications for Prevention Programs: Summary of a Workshop” at NAP.edu [Internet].

